# Changes in body mass index, weight, and waist-to-hip ratio over five years in HIV-positive individuals in the HIV Heart Aging Study compared to the general population

**DOI:** 10.1007/s15010-023-02009-8

**Published:** 2023-03-17

**Authors:** Laven Mavarani, Sarah Albayrak-Rena, Anja Potthoff, Martin Hower, Sebastian Dolff, Stefanie Sammet, Felix Maischack, Dirk Schadendorf, Börge Schmidt, Stefan Esser, on behalf of HIV HEART AGING Study

**Affiliations:** 1 Institute for Medical Informatics, Biometry and Epidemiology (IMIBE), University Hospital Essen, University Duisburg-Essen, Essen, Germany; 2 Department of Dermatology and Venereology, HIV Outpatient Clinic, University Hospital Essen, University Duisburg-Essen, Essen, Germany; 3 Interdisciplinary Immunological Outpatient Clinic, Center for Sexual Health and Medicine, Department of Dermatology, Venereology and Allergology, Ruhr University Bochum, Bochum, Germany; 4 Department of Pneumology, Infectious Diseases and Internal Medicine, Klinikum Dortmund, Hospital University Witten/ Herdecke, Dortmund, Germany; 5 Department of Infectious Diseases, University Hospital Essen, University Duisburg-Essen, Essen, Germany; 6 Institute for Translational HIV Research, University Hospital Essen, University Duisburg-Essen, Essen, Germany

**Keywords:** HIV, Waist-to-hip ratio, Weight gain, Risk factors, Body mass index, Obesity

## Abstract

**Purpose:**

Overweight and obesity have increased in people living with HIV (PLH). Our study evaluated weight, body-mass-index (BMI), and waist-to-hip ratio (WHR) change over 5 years of follow-up in PLH compared to the general population.

**Methods:**

HIV-positive participants in the HIV Heart Aging (HIVH) study were matched 1:2 by age and sex with HIV-negative controls of the population-based Heinz Nixdorf Recall (HNR) study. Both studies were recruited in the German Ruhr area. The association between HIV and weight, BMI, and WHR changes was examined using linear regression. Regression models were adjusted for parameters potentially affecting weight gain.

**Results:**

The matched HIVH and HNR participants (*N* = 585 and *N* = 1170, respectively; 14.7% females) had a mean age of 55 years at baseline. Despite the lower baseline weight (− 6 kg, 95% CI − 7.46 to − 4.59), the linear regression showed greater absolute and relative weight and BMI increases after 5 years in HIVH compared to HNR. Adjusting the linear regression models for smoking amplified that HIVH had a higher absolute and relative weight difference of 0.7 kg or ~ 1% compared to HNR after 5 years (95% Cl 0.1 to 1.3 and 0.2 to 1.6, respectively). Adjusting for HDL, LDL, systolic blood pressure, and diabetes mellitus did not affect the results.

**Conclusions:**

PLH had lower weight than the general population at baseline and after 5 years, but experienced greater increases in body weight after 5 years. WHR change after 5 years was lower in PLH compared to the general population, despite a higher WHR at baseline.

**Supplementary Information:**

The online version contains supplementary material available at 10.1007/s15010-023-02009-8.

## Introduction

The prevalence of obesity and obesity-related comorbidities have been increasing worldwide over the last several decades [1, 2], including in people living with HIV (PLH) [3, 4]. However, in some cohort studies, PLH still have lower rates of obesity than age-matched HIV-negative controls [5]. Weight gain in PLH and the general population is multifactorial and associated with demographic factors, diet, exercise habits, psychological health, other comorbidities, and co-medications. Additionally, in PLH HIV disease parameters and the composition of antiretroviral therapies influence weight gain [6–10]. HIV-associated wasting declined with the introduction of antiretroviral therapy (ART). Weight gain after initiation of ART has been associated with a reduced risk of mortality in individuals with underweight and individuals with normal weight [11]. While this increase in body weight with the initiation of ART is seen as a “return to health”, especially in patients with advanced disease [12], excessive weight and body-mass-index (BMI) increase in some individuals receiving contemporary ART may lead to obesity. So far, it has not been fully elucidated whether weight gain in aging PLH over time is substantially different from weight gain observed in the general population. Overweight and obesity are associated with several health issues, such as diabetes mellitus type II and cardiovascular disease [13].

Apart from body weight, BMI is generally used for evaluating overweight or obesity in the clinical setting, calculated from body weight and height. There are four categories of BMI that were established by the WHO in 1993: underweight, normal, overweight, and obese [14]. BMI increase can be associated with insulin resistance (IR), hyperglycemia, and new-onset diabetes mellitus type II, [15–17]. Initiating ART with or switching to second generation integrase strand transfer inhibitors (INSTIs) and/or tenofovir alafenamide (TAF) has shown to be associated with more pronounced weight and BMI increases [8–10, 18–20]. Baseline ART was a predictor of post-switch weight gain. Using tenofovir disoproxil (TDF) was associated with reduced weight gain [21].

Nevertheless, BMI or body weight alone as an indicator of obesity does not differentiate between body lean mass and body fat mass since fat mass is not measured directly [22, 23]. Studies have shown that waist circumference correlates better with body fat than BMI, and thus, waist circumference alone or in combination with BMI has a stronger relation to specific health outcomes than BMI alone [24]. Other studies have shown associations between waist-to-hip ratio and cardiovascular events, metabolic risk factors, and death and suggested that it is superior to waist circumference alone, because it includes the hip circumference, too [25–28].

Due to the various limitations of current analyses, more data and efforts are necessary to decipher the driving factors behind weight gain in PLH. It is important to define whether the general risk factors for weight gain and increased BMI have a similar impact on PLH compared to the general population. Most importantly, longitudinal data from clinical routines over more than one or two years are needed to describe the effects of weight changes.

The aim of our study was to compare changes in weight, BMI, and waist-to-hip-ratio (WHR) over a 5-year-follow-up period between PLH from the HIV Heart Aging (HIVH) cohort [29] and HIV-negative participants from the Heinz-Nixdorf-Recall (HNR) study [30], a cohort study from the same geographical area in Germany (Ruhr area) as the HIV Heart Aging study.

## Methods

### Study population

The HIVH study is an ongoing prospective, observational, multicenter cohort study in the German Ruhr Area which started in 2004. The study population has been recruited to assess the incidence and clinical course of cardiovascular diseases (CVD) in PLH. Up to now, 1807 individuals (1533 male, 274 female), ranging from 18 to 79 years of life at baseline, have been recruited within this cohort. The HIVH study has been previously described in detail [29]. In brief, HIV-positive outpatients from specialized HIV-care units within the German Ruhr area who were at least 18 years of age and had stable disease status throughout the last four weeks were included in the cohort. Follow-up examinations were performed every 2.5 years. The cohort has been registered at Clinical Trails (NCT04330287) and has been approved by the local ethics committee of the University Duisburg-Essen (14-5874-BO) [31]. All participants gave written informed consent. The standardized examinations contained a medical examination, including the assessment of body composition like weight (in underclothes), standing height, and WHR. Also lipodystrophy, lipoatrophy and lipohypertrophy were assessed by reporting the subjective impression of the physician and/or the study participant. Moreover, the medical history of the HIVH participants was assessed. Blood was drawn for comprehensive laboratory tests, where levels of total cholesterol (TC), high-density lipoprotein (HDL) cholesterol, low-density lipoprotein (LDL) cholesterol, viral load, and CD4 count were quantified. Plasma and serum samples were stored at – 80 °C. Further details regarding the recruitment and baseline examination of the HIVH study have been published previously [29].

The HNR study is a single-center study that included 4814 participants between 45 and 75 years of age in the Ruhr area, Germany, between 2000 and 2003. It has been designed to investigate the predictive value of novel risk markers for CVD. Participants were selected randomly from residence registers of the Ruhr area. A detailed description of the HNR study design, recruitment, and risk factor assessment has been described elsewhere [30]. All participants have given written consent and the study was approved by the local ethics committee. On an annual basis, questionnaires regarding the current stage of health were sent out to all participants and in parallel death certificates were screened regularly. At baseline, all participants included in the HNR study underwent a physical examination including weight, height, WHR, blood pressure assessment, their medical history assessed, and blood samples were drawn by comprehensive laboratory tests. Plasma and serum samples were stored at – 80 °C. Blood lipids (TC, HDL, and LDL) were quantified. Body height was measured in a standardized way. BMI was calculated based on standardized weight (in underclothes) and body height measures.

### Exclusion criteria

To harmonize the age range between the studies, HIVH participants were selected by their most recent visit (further called “baseline”) and corresponding 5-year follow-up with available weight and BMI measurements (baseline range: 2006–2016). By using this kind of backward selection, the average age of the HIVH participants was higher than when starting from the original baseline. Moreover, the backward selection allows a better reflection of current medical care and contemporary antiretroviral treatment. The control group with HIV-negative persons from the HNR study included participants starting from an age of 45 years, participants younger than 45 years from the HIVH study were excluded in a second step. In HNR the original baseline at enrolment in 2000–2003 was used in order to prevent an even older population, and thus prevent more participants from being excluded before matching HIVH and HNR. In HIVH pregnant women were excluded at the enrolment of the study, while in HNR there were no pregnancies due to the high age of the female participants. Afterward, the remaining participants from the HIVH and HNR studies were matched 1:2 by age and sex. In total, there were up to 585 PLH from the HIVH study and 1170 HIV-negative participants from the HNR study included in the analysis. The average follow-up time was 5.13 years in HNR and 5.22 years in HIVH. The flowchart of the population is displayed in Online Resource 1.

### Statistical analysis

We summarized the general characteristics (age, sex), anthropomorphic parameters (weight, BMI, WHR), potential weight gain affecting parameters (smoking state), and parameters affected by weight gain (blood pressure, diabetes mellitus type II, total cholesterol, LDL, HDL), and the HIV-specific parameters (ethnicity, mode of HIV transmission, immunological and clinical HIV categories, CD4/CD8 ratio, viral load, duration of HIV infection, and ART medication) descriptively. Then, the absolute and relative differences in weight, BMI, waist circumference, hip circumference, and WHR after 5 years were calculated and standardized for 5 years using the exact days between the date of follow-up and the date of the baseline. Absolute and relative differences were presented as histograms. Sankey bar charts were created, showing the shift in BMI and WHR groups during 5 years in the HIVH and HNR analysis population. Therefore, BMI and WHR were categorized into groups according to recommended WHO cutoffs [32, 33]. For the BMI categorization, underweight was specified as a BMI < 18.5 units. Normal and overweight BMI was defined between 18.5 and < 25 and 25 to < 30 BMI units, respectively. BMI ≥ 30 units was categorized as having obesity. According to the WHO categorization, females with a WHR of ≥ 0.85 cm and males with a WHR of ≥ 0.9 cm have substantially increased risk of metabolic complications.

In the additional information given by the Online Resources, the analyzed participants were stratified by relative weight change cutoffs (weight change ≤ − 10%, weight change from > − 10 to < 10%, weight change ≥ 10%) and the general characteristics and previously described parameters were summarized. The absolute differences in blood pressure, diabetes mellitus type II, total cholesterol, LDL, HDL, weight, BMI, waist and hip circumference, and WHR within 5 years were calculated and standardized using the same method as above. An increase or decrease in the calculated change was visualized by a positive or negative algebraic sign.

Linear regression models were calculated to assess the association of HIV (HIVH participants vs. HNR participants) with weight, BMI, and WHR at baseline. Further linear regression models investigated the associations between differences in weight, BMI, and WHR within 5 years and the presence of an HIV infection. Beta estimates, the corresponding 95% confidence limits (Cl), and *p* values were reported. Then, the linear regression models for weight, BMI, WHR differences within the time were adjusted for smoking, a potential confounder for weight gain. The same linear regression models were then repeated by adjusting for other parameters affected by weight and body composition, like systolic blood pressure, diabetes mellitus type II, LDL, and HDL in addition to smoking. The calculated beta estimates in the linear regression models represent the group differences between HIVH and HNR in the respective outcome.

All analyses were performed using SAS software, version 9.4 (SAS Institute Inc., Cary, NC, USA.).

## Results

Within the analysis population, the matched participants in the HIVH and HNR study were predominantly male (~ 85%), with a mean age of 55 years. Further baseline characteristics of the HIVH and HNR study participants are shown in Table 1. Parameters such as blood lipids, weight, BMI, waist circumference, and hip circumference were lower in HIVH at baseline than in HNR. The prevalence of diabetes mellitus type II was also lower in HIVH compared to the HNR cohort (9.3% vs. 13.5% at baseline, respectively). The percentage of smoking participants in HIVH was higher than in HNR (44.5 vs. 28.8% at baseline, respectively). The absolute and relative differences after 5 years of weight, BMI, and waist-to-hip ratio (WHR) are also shown in Table 1. While HIVH participants had lower weight and BMI than the HNR participants at baseline, relative weight and BMI gain after 5 years was slightly higher in PLH (weight (kg):1.61 ± 6.7 vs. 1.00 ± 5.13; BMI (kg/m^2^): 0.59 ± 2.20 vs. 0.46 ± 1.72). However, WHR was higher in HIVH than HNR at baseline. The absolute WHR difference remained unchanged after 5 years in HIVH, but increased in HNR (0 ± 0.08 vs. 0.02 ± 0.06 units, respectively). The relative WHR difference after 5 years shows a decrease in WHR in PLH, while WHR in the general population increases (− 0.04 ± 8.12 vs. 2.08 ± 6.2%, respectively).

**Table 1:** HIVH and HNR characteristics (matched by age and sex) for baseline and 5-year follow-up

		Baseline				5-Year follow-up			
		HIVH		HNR		HIVH		HNR	
		*N*	*n* (%)/Mean ± SD	*N*	*n* (%)/Mean ± SD	N	*n* (%)/Mean ± SD	*N*	*n* (%)/Mean ± SD
Sex	Female	585	86 (14.7%)	1170	172 (14.7%)	585	86 (14.7%)	1170	172 (14.7%)
Age	[years]	585	54.9 ± 6.8	1170	54.9 ± 6.8	585	60.1 ± 6.8	1170	60.0 ± 6.8
Blood pressure	Systolic [mmHg]	516	139.3 ± 19.1	1168	133.6 ± 19.1	474	137.4 ± 18.3	1168	134.7 ± 19.2
	Diastolic [mmHg]	516	84.0 ± 11.6	1168	83.6 ± 10.8	474	81.9 ± 10.9	1168	81.1 ± 10.8
Diabetes mellitus type II	Yes	559	52 (9.3%)	1170	158 (13.5%)	82	82 (14.0%)	1170	228 (19.5%)
Total cholesterol	[mg/dl]	578	212.5 ± 42.5	1166	225.0 ± 37.6	556	215.8 ± 46.5	1167	220.6 ± 40.9
LDL	[mg/dl]	522	130.1 ± 38.2	1162	143.8 ± 35.3	526	136.2 ± 41.7	1167	130.9 ± 34.3
HDL	[mg/dl]	543	50.0 ± 15.8	1165	52.8 ± 15.2	522	50.9 ± 15.9	1167	54.6 ± 14.4
weight	[kg]	585	77.8 ± 14.5	1170	84.1 ± 14.4	585	79.7 ± 15.7	1170	85.1 ± 14.8
BMI	[kg/m^2^]	585	25.1 ± 4.2	1170	27.7 ± 4.0	585	25.8 ± 4.8	1170	28.2 ± 4.2
Waist-to-hip ratio		472	0.98 ± 0.07	1169	0.95 ± 0.08	468	0.98 ± 0.07	1170	0.97 ± 0.08
Smoking	Yes	490	213 (43.5%)	1170	337 (28.8%)	520	206 (39.6%)	1168	286 (24.5%)
Std. abs. weight difference/5 years	[kg]					585	1.61 ± 6.37	1170	1 ± 5.13
Std. rel. weight difference/5 years	[%]					585	2.18 ± 8.04	1170	1.33 ± 5.98
Std. abs. BMI difference/5 years	[kg/m^2^]					585	0.59 ± 2.20	1170	0.46 ± 1.72
Std. rel. BMI difference/5 years	[%]					585	2.4 ± 8.33	1170	1.79 ± 6.05
Std. abs. waist-to-hip ratio difference/5 years						418	0 ± 0.08	1169	0.02 ± 0.06
Std. rel. waist-to-hip ratio difference/5 years	[%]					418	− 0.04 ± 8.12	1169	2.08 ± 6.2

HIV-specific characteristics are shown in Table 2. Among the HIVH participants, the mean age at the diagnosis of the HIV infection was ~ 41 years, with men having sex with men (MSM) as the main HIV transmission risk category (61%). At baseline, more than one-third had at least one documented AIDS-defining event in their medical history. The vast majority of the participants (> 99%) received ART at baseline. During the observation period of five years, the use of antiretroviral drug classes was changing: There was an increase in the use of INSTIs (14.2–44.4%), while the use of NNRTIs (47.4–39.1%) and PIs decreased (47.4–29.0%). Among the NRTIs, at baseline only 1% of the PLH used tenofovir alafenamide (TAF) in their ART regimen. After 5 years, this had increased to 40.1%. Also, the use of single tablet regimens increased (16.5% to 40.6%). CD4/CD8 ratio improved over the 5 years of observation among HIVH (0.8 ± 0.43–1.01 ± 1.98). The proportion of participants with HIV RNA below the level of detection (< 50 copies/ml) was 93.3% at baseline and did not change within the five years of follow-up (93.9%). Lipodystrophy syndrome is divided into lipodystrophy, lipoatrophy and lipohypertrophy and were acquired in HIVH. At baseline 22.8% of the PLH from HIVH had lipodystrophy, and 22% had lipoatrophy. Furthermore, 10.3% of the HIVH participants had lipohypertrophy. During the observation time of 5 years, the percentages of lipoatrophy and lipohypertrophy reduced to 20.7% and 8.4%, respectively. Lipodystrophy increased by less than 1%.

**Table 2:** HIV-specific characteristics in the matched analysis population of HIVH at baseline and after 5 years

		HIVH baseline		HIVH after 5 years	
		*N*	*n* (%)/Mean ± SD	*N*	*n* (%)/MEAN ± SD
Age at HIV-infection	[years]	573	40.7 ± 9.3	573	40.7 ± 9.3
Ethnicity	Caucasian	585	539 (92.1%)	585	539 (92.1%)
	African	585	9 (1.5%)	585	9 (1.5%)
	Asian	585	5 (0.9%)	585	5 (0.9%)
	Other	585	32 (5.5%)	585	32 (5.5%)
Way of HIV Transmission	MSM	557	353 (63.4%)	557	353 (63.4%)
	Hetero	557	126 (22.6%)	557	126 (22.6%)
	Intravenous drug use	557	36 (6.5%)	557	36 (6.5%)
	Transfusion	557	10 (1.8%)	557	10 (1.8%)
	Epidemic area	557	31 (5.6%)	557	31 (5.6%)
	Other	557	1 (0.2%)	557	1 (0.2%)
HIV category (immunological)	HIV I	557	25 (4.5%)	584	17 (2.9%)
	HIV II	557	236 (42.4%)	584	242 (41.4%)
	HIV III	557	296 (53.1%)	584	325 (55.7%)
HIV category (clinical)	HIV A	557	176 (31.6%)	584	164 (28.1%)
	HIV B	557	184 (33.0%)	584	198 (33.9%)
	HIV C/AIDS	557	197 (35.4%)	584	222 (38.0%)
CD4/CD8 ratio		579	0.8 ± 0.43	580	1.01 ± 1.98
Viral load	[copies/ml]	585	902.4 ± 11,451.0	584	435.4 ± 6961.7
Viral load < 50 copies/ml	Yes	585	546 (93.3%)	585	549 (93.9%)
Under ART medication	Yes	570	569 (99.8%)	576	576 (100%)
Duration of HIV infection	[years]	573	14.1 ± 6.7	573	19.4 ± 6.7
ART medication categories	NRTIs	570	538 (94.4%)	576	525 (91.1%)
	NRTIs: TAF	570	5 (0.9%)	576	231 (40.1%)
	NNRTIs	570	270 (47.4%)	576	225 (39.1%)
	INSTIs	570	81 (14.2%)	576	256 (44.4%)
	PIs	570	270 (47.4%)	576	167 (29.0%)
	Boost	570	192 (33.7%)	576	174 (30.2%)
	Entry inhibitor	570	16 (2.8%)	576	18 (3.1%)
	Combination medication	570	405 (71.1%)	576	272 (47.2%)
	Single tablet regime	570	94 (16.5%)	576	234 (40.6%)
Lipodystrophy	Yes	549	125 (22.8%)	584	137 (23.5%)
Lipoatrophy	Yes	551	121 (22.0%)	585	121 (20.7%)
Lipohypertrophy	Yes	551	57 (10.3%)	584	49 (8.4%)

Histograms for the absolute and relative weight, BMI, and WHR differences after 5 years in the two study populations are shown in Online Resource 2. The histograms for weight and BMI (Online Resource 2 a + b and c + d, respectively) showed a wider differences distribution after 5 years in HIVH than in HNR. For WHR the histograms reflected a reduction in WHR in HIVH in comparison to HNR (Online Resource 2 e + f).

**Fig. 1:**
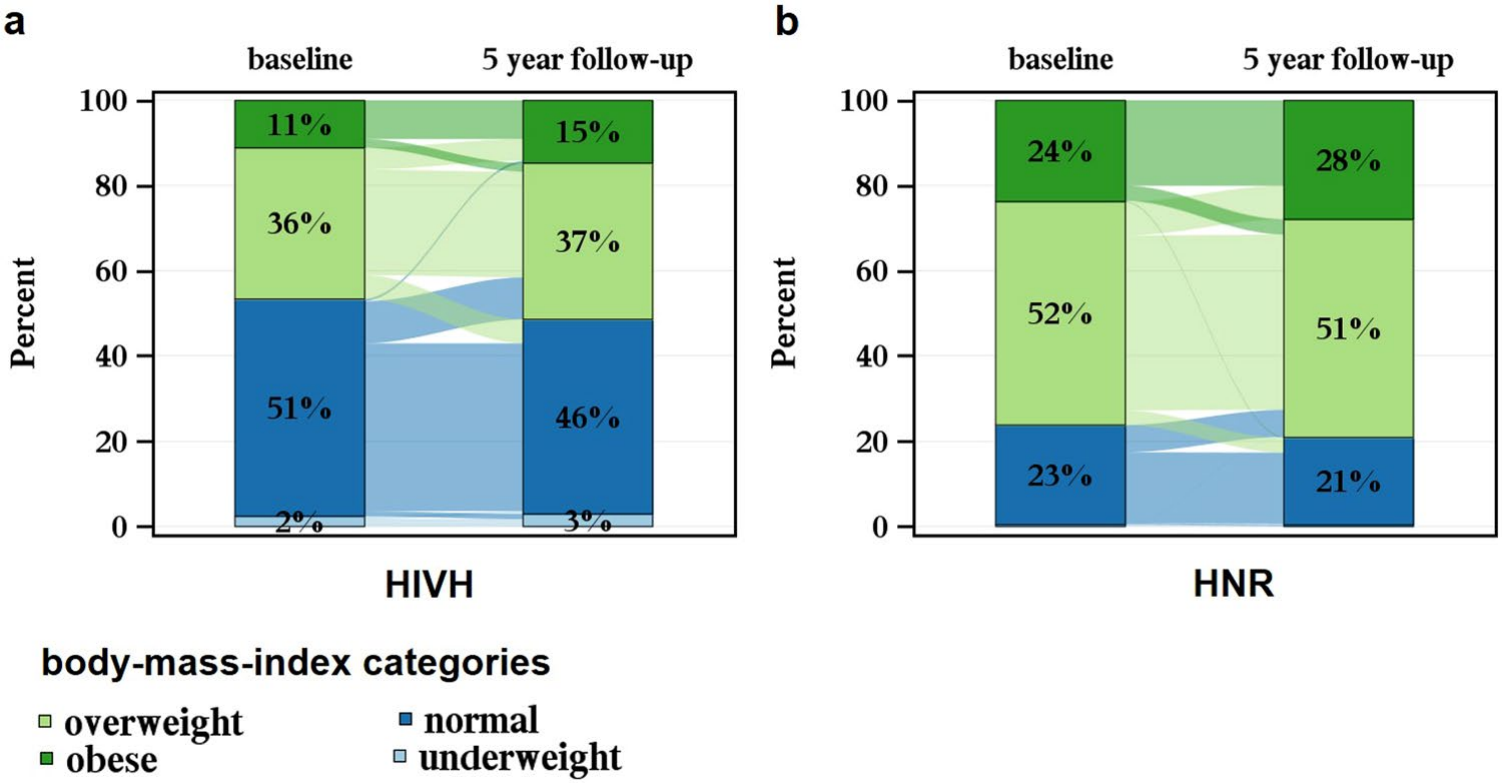
Shift in body-mass-index categories during 5 years—**a** HNR (*n* = 585) vs. **b** HIVH (*n* = 1170) matched for age and sex at baseline

Sankey bar diagrams in Fig. 1 visualize the shift between BMI categories during 5 years in the two study populations. HIVH and HNR differed regarding their percentage BMI categories distributions at baseline. While the HIVH study had more participants with a normal BMI (51%) and 2% participants in the underweight category, the HNR study had only 23% participants with normal BMI and no underweight participants. More than 50% of the HNR participants had overweight, and almost one-quarter had obesity at baseline. 36% of the HIVH participants were in the overweight BMI category, and 11% had obesity. After 5 years, the changes within the BMI categories were small in both HIVH and HNR, and we observed similar trends regarding the group shifts. The normal BMI category decreased after 5 years (HIVH: from 51 to 46% vs. HNR from 23 to 21%), while the group with obesity increased during the observation period (HIVH 15% vs. HNR 28%). BMI changes of more than one category rarely occurred (< 1%). Despite higher weight gains over time, the proportion of overweight and obesity in the HIVH cohort was still lower compared to the HNR participants. Excessive weight gain or loss of 10% or more occurred in ~ 20% of the participants in HIVH and ~ 10% of participants in HNR (Online Resource 3). 13.5% of the HIVH vs 6.2% of the HNR participants had ≥ 10% weight gain after 5 years, while 5.8% in the HIVH vs 2.8% in the HNR cohort lost ≥ 10% of their weight.

**Fig. 2:**
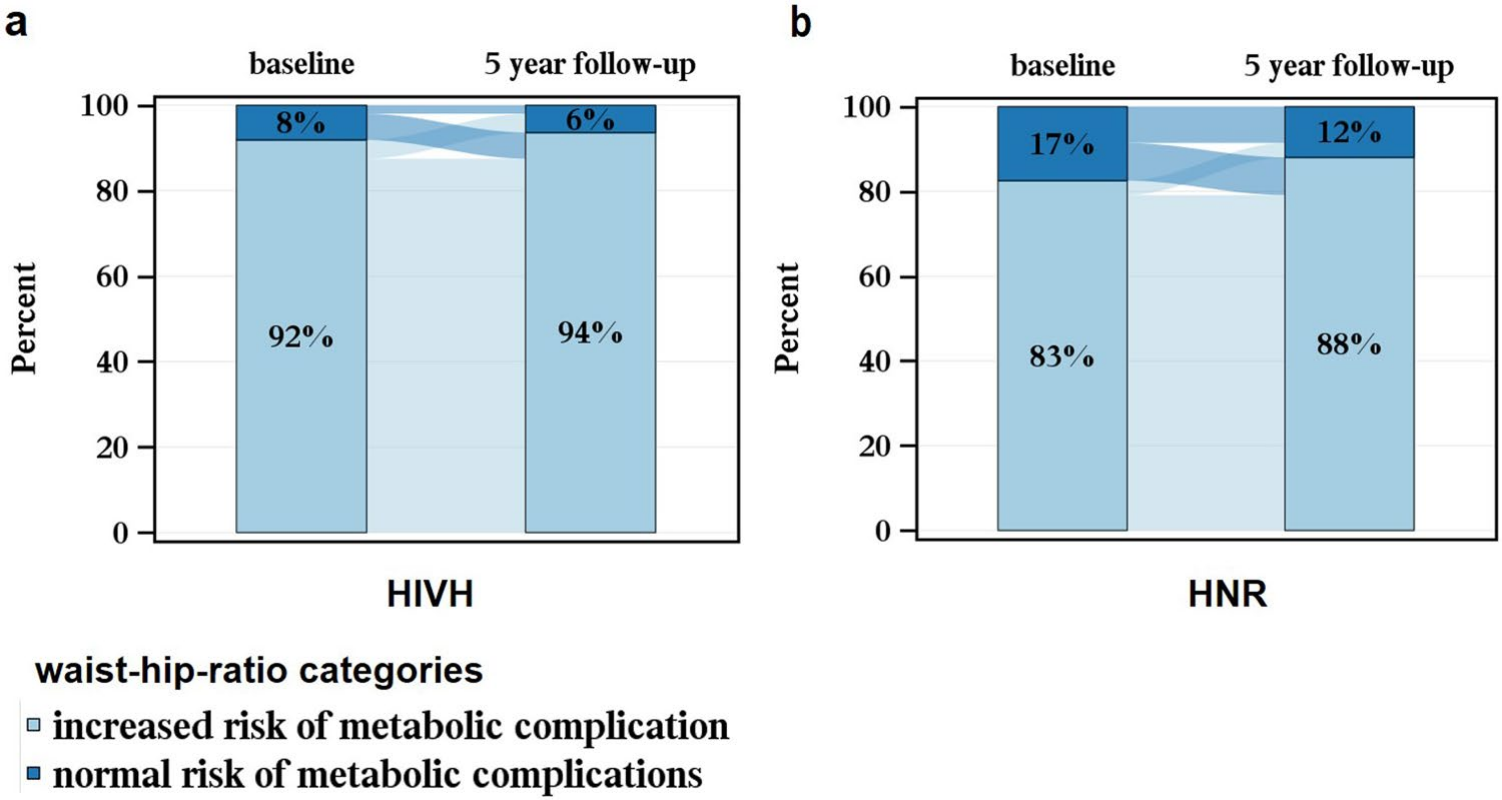
Shift in waist-hip-ratio categories during 5 years—**a** HNR (*n* = 585) vs. **b** HIVH (*n* = 1170) matched for age and sex at baseline

The Sankey diagrams in Fig. 2 show the shift in WHR categories over 5 years between HIVH and HNR using the categorization of the World Health Organization (WHO) Expert Consultation on Waist Circumference and Waist–Hip Ratio. They revealed that in both study groups the majority of the participants have an “increased risk of metabolic complications” at baseline according to their WHR (> 80%), which increased further after 5 years. The percentage of the HNR study participants with “normal risk of metabolic complications” according to their WHR changed from 17 to 12% over 5 years. “Normal risk for metabolic complications” in HIVH changed from 8 to 6%, therefore, a percentage twice as high as in HNR had an “increased risk of metabolic complications”, despite the lower baseline and post-5 years weight in HIVH.

The results of the linear regression models for assessing associations of HIV status with weight, BMI, and WHR at baseline and their changes after 5 years as outcome are shown in Table 3. A group difference in weight between HIVH and HNR at baseline of ~ 6 kg reflects the lower body weight in HIVH participants at baseline (95% Cl − 7.5 to − 4.6). The absolute change in weight after 5 years of follow-up was 0.61 kg (95% Cl 0.1 to 1.2), and the relative change was 0.85% (95% Cl 0.2 to 1.5) higher in HIVH compared to HNR. When adjusting for smoking the relative and absolute weight difference after five years was even more distinct (0.71 kg and 0.93%, respectively), demonstrating the influence of smoking on weight gain and the differences in smoking status between HIVH and HNR.

**Table 3:** Group difference between PLH (HIVH) and the general population (HNR) for the anthropometric outcomes weight, BMI, and WHR at baseline and absolute and relative differences after 5 years in linear regression models

Anthropometric outcomes	1:2 matched by age and sex					1:2 matched by age and sex adjusted for smoking				1:2 matched by age and sex adjusted for smoking. sys. BP. DM. HDL. LDL			
	β	95% CL			*P* value	β	95% CL		*P* value	β	95% CL		*P* value
		LCL		UCL			LCL	UCL			LCL	UCL	
	Missings = 0					Missings = 95				Missings = 139			
Weight at baseline [kg]	− 6.02	− 7.46		− 4.59	< 0.0001	–	–	–	–	–	–	–	–
Abs. weight diff. after 5 years [kg]	0.61	0.06		1.16	0.0307	0.71	0. 13	1.29	0.016	0.67	0.06	1.27	0.032
Rel. weight diff. after 5 years [%]	0.85	0.18		1.51	0.0133	0.93	0.24	1.63	0.009	0.91	0.17	1.64	0.015
	Missings = 0					Missings = 95				Missings = 139			
BMI at baseline [units]	− 2.56	− 2.96		− 2.15	< 0.0001	–	–	–	–	–	–	–	–
Abs. BMI diff. after 5 years [units]	0.13	− 0.06		0.31	0.1849	0.16	− 0.03	0.36	0.102	0.15	− 0.06	0.35	0.168
Rel. BMI diff. after 5 years [%]	0.61	− 0.08		1.29	0.0827	0.72	0.01	1.43	0.048	0.68	− 0.07	1.43	0.077
	Missings = 168					Missings = 179				Missings = 207			
WHR at baseline [cm]	0.03	0.02		0.04	< 0.0001	–	–	–	–	–	–	–	–
Abs. WHR diff. after 5 years [cm]	− 0.02	− 0.03		− 0.01	< 0.0001	− 0.02	− 0.03	− 0.01	< 0.0001	− 0.02	− 0.03	− 0.01	< 0.0001
Rel. WHR diff. after 5 years [%]	− 2.12	− 2.88		− 1.37	< 0.0001	− 1.90	− 2.66	− 1.15	< 0.0001	− 2.18	− 2.97	− 1.38	< 0.0001

*abs.* absolute, *rel.* relative, *β* parameter estimate, *95% CL* 95% confidence limit, *LCL* lower confidence limit, *UCL* upper confidence limit, *BP* blood pressure, *DM* diabetes mellitus type II

HIVH had a 2.6 kg/m^2^ lower baseline BMI compared to HNR (95% Cl − 3.0 to − 2.2). The absolute change in BMI after 5 years of follow-up was 0.13 kg/m^2^ (95% Cl − 0.1 to 0.3) higher in HIVH compared to HNR, while the relative change was 0.6% kg/m^2^ (95% Cl − 0.1 to 1.3) higher in HIVH compared to HNR. After adjusting for smoking beta estimates were slightly higher.

We observed a difference in WHR between HIVH and HNR at baseline of ~ 0.03 units (95% Cl 0.02 to 0.03). The absolute and relative WHR change after 5 years of follow-up was lower by 0.02 units and 2.1% in HIVH compared to HNR, respectively. Beta estimates differed only slightly after adjustment for smoking.

After adjusting all regression models for parameters potentially affecting body weight change (i.e., diabetes mellitus type II, LDL, HDL, and systolic blood pressure) in addition to smoking, beta estimates for assessing the strength of association between HIV status and differences in anthropometric measures over 5 years were not affected, demonstrating that the observed differences between HIVH and HNR were independent of the parameters adjusted for (Table 3). This was also reflected by the results displayed in Online Resource 4. Here, absolute differences of parameters potentially affected by body weight change after 5 years were presented stratified by different categories of relative weight change. No strong differences in these parameters were observed between HIVH and HNR across weight change categories.

## Discussion

This study compared body weight, BMI, and WHR in PLH and individuals from the general population to show the changes in these parameters after 5 years of follow-up. We observed lower baseline body weight and BMI in PLH compared to the general population like in another trial with age-matched controls [5]. The absolute and relative differences after 5 years in weight and BMI were slightly higher in PLH as well. Smoking has been correlated with lower body weight in several studies [34]. After adjusting for the potential confounder “smoking”, the linear regression models revealed the underlying effect: HIV is associated with weight gain, with an increase of 0.7 kg weight (relative weight gain of ~ 1%) within 5 years in PLH compared to the general population. Other studies demonstrated similar findings, although the amount of weight gain was variable [5, 35–37]. This effect is also observed for BMI, although not as strongly as for weight (absolute/relative BMI gain in PLH by 0.15 units/0.7%). According to prior studies, living with HIV has been associated with lower weight and body constitution at the time of diagnosis [5, 38, 39]. Due to improved ART medication and effective suppression of the virus, the weight gain is observed over time in ART-naive PLH and is considered part of the normal return to health following successful antiretroviral treatment [3, 40]. The weight gain in the first year after ART initiation was associated with survival benefits among initially underweight and normal-weight patients [41]. Prior studies could not clearly show if weight gain in PLH over time was substantially different from the general population. Excessive weight change (≥ 10%) was twice as frequent in HIVH compared to the HNR cohort after 5 years. Thereby, excessive weight gain was twice as frequent compared to excessive weight loss in both cohorts. Both excessive weight gain and weight loss occurred twice as frequently in HIVH participants compared to HNR participants during the observation period. Recently, a study by Garcia et al. showed that weight gain was primarily observed in PLH with BMI < 25 at diagnosis, suggesting that baseline weight, time since HIV diagnosis, and past weight history should be taken into account [5]. The comparison of PLH with the general population of the same age and the same geographic area shows that the general population in the German Ruhr area already has high rates of overweight and obesity, which are associated with health issues like diabetes mellitus type II and cardiovascular diseases [13]. Our results show that the proportion of overweight and obesity in PLH was still much lower than in the general population, suggesting that the BMI shift among PLH reflected the “return-to-health” respectively “coming-back-to-normal” effects of the ART. The observed BMI shifts after 5 years as a result of the weight gain in PLH shown in the Sankey bar charts might present the aligning of the BMI of PLH with the general population, but does not inevitably mean a “return to health”. In the cohort of the US veterans, the same trends in veterans living with HIV compared to HIV-negative veterans were observed during a 5-year follow-up period but the weight gain was described as transient [5].

The BMI allows the classification of patients as having overweight or having obesity and the comparability across different populations and areas. But the BMI is only of limited use for predicting cardio-metabolic risk because relevant factors such as muscle mass and fat distribution are not taken into account. Intra-abdominal visceral adiposity is associated with an elevated cardio-metabolic risk but does not always present with an increased BMI [42]. An Italian study has even demonstrated improved metabolic profiles in PLH in recent years compared with previous, despite increasing weight and BMI [43]. The WHR measures central adiposity more accurately and might be a more appropriate predictor of cardio-metabolic risk [42]. WHR in HIVH was higher at baseline than in participants from HNR representing the general population. HIV infection, some protease inhibitors, and first-generation NRTIs were associated with lipodystrophy syndrome. Metabolic syndrome, insulin resistance, and liver steatosis were common in PLH and associated with abdominal obesity [40, 44], which might explain the less favorable WHR in HIVH vs. HNR participants. The absolute and relative WHR differences within 5 years were lower in HIVH. Thus, increases in WHR were less pronounced in HIVH participants, which may reflect the already high WHR in HIVH compared to HNR. Moreover, we observed a decrease in lipohypertrophy as part of the lipodystrophy syndrome, which also reflects the decrease in WHR change after 5 years in PLH. The gain of fat in PLH treated with modern ART regimens including INSTI is generalized in most patients and not defined as lipodystrophy [40]. Previous studies observed no weight gain in PLH with baseline CD4 > 200 cells/µL and viral load < 100,000 copies/mL [45]. In our analysis, the majority of the participants in HIVH (> 93%) had a viral load of < 50 copies/ml and therefore the association with viral load was not analyzed here. We could, however, observe that younger age was associated with more weight gain [5] when stratifying for weight gain differences > 10% (see Online Resource 5).

### Strength and limitations

The main strength of this study is that both cohorts were recruited from the same geographical area in Germany. Despite the lack of ethnicity information from the participants of HNR, we can assume that the distribution of ethnicities might be similar.

Unfortunately, we were unable to use the full potential of the HIVH study, since HNR participants are older, so younger HIVH participants had to be excluded. With only 15% of female participants in HIVH in our analysis, weight gain among females cannot be assessed. We also observed a large difference in smokers between HIVH and HNR, which suggests that there might be a larger set of covariates that are not assessed that are associated with weight and weight change. Socioeconomic position and lifestyle need to be analyzed in future studies. Another limitation might be that we only included living participants in our analysis population. Furthermore, since we compare PLH with the general population, we did not account for the type of ART medication in the PLH group in this analysis. Furthermore, other co-medications that affect weight gain or weight loss, such as lipid-lowering drugs and antidiabetics, were not accessed in a standardized way and therefore were not taken into account in the comparison of HNR and HIVH. Another limitation regarding the excluded participants with only one weight/BMI measurement in our analysis might be that we do not know if morbidity and mortality in these participants is the reason for their lost-to-follow-up, which is potentially associated with a higher risk profile in the excluded participants.

## Conclusion

While PLH had lower weight and BMI than the general population, weight, and BMI gain after 5 years was slightly higher in PLH. This effect is more pronounced after adjusting for smoking, which has a major impact on weight gain. This increasing weight difference after 5 years in PLH compared to the general population could be the result of the “return-to-health”, respectively, “coming-back-to-normal” effects of the treatment with ART. Moreover, we observed that WHR differences after 5 years are lower in PLH due to a higher WHR at baseline compared to the general population and thus increased risk of metabolic and cardiovascular complications according to WHO recommendations. During the 5 years of observation the lipodystrophy syndrome did not increase, but rather trends toward a decrease, which also affects WHR in PLH. Longitudinal data from clinical cohorts over more than one or two years are needed to describe the effects of weight changes in PLH.

## Supplementary Information

Below is the link to the electronic supplementary material.

Supplementary file1 (DOCX 435 KB)

Supplementary file2 (PDF 233 KB)

## Data Availability

Due to data security reasons (i.e., data contain potentially participant identifying information), the HIV Heart Aging Study does not allow sharing data as a public use file. However, others can access the data used upon request, which is the same way the authors of the present paper obtained the data. Data requests can be addressed to: Stefan.Esser@uk-essen.de. Due to data security reasons (i.e., data contain potentially participant identifying information), the Heinz Nixdorf Recall Study does not allow sharing data as a public use file. However, others can access the data used upon request, which is the same way the authors of the present paper obtained the data. Data requests can be addressed to: recall@uk-essen.de.
