# Supplementary material for: Changes in body mass index, weight, and waist-to-hip ratio over five years in HIV-positive individuals in the HIV Heart Aging Study compared to the general population
Source: Infection. 2023 Mar 17;51(4):1081–91. doi: 10.1007/s15010-023-02009-8 (PMC10352154; doi:10.1007/s15010-023-02009-8)
Supplement: Supplementary file 1 — Supplementary file1 (DOCX 435 KB) [file 15010_2023_2009_MOESM1_ESM.docx]

**Changes in body mass index, weight, and waist-to-hip ratio over five years in HIV-positive individuals in the HIV Heart Aging Study compared to the general population**

**Infection**

**Authors:**

Laven Mavarani^1^, Sarah Albayrak-Rena^2^, Anja Potthoff^3^, Martin Hower^4^, Sebastian Dolff^5^, Stefanie Sammet^2^, Felix Maischack^2^, Dirk Schadendorf^2^, Börge Schmidt^1^, Stefan Esser^2,6^ on behalf of HIV HEART AGING Study

**Affiliations:**

^1^ Institute for Medical Informatics, Biometry and Epidemiology (IMIBE), University Hospital Essen, Essen, Germany

^2^ Department of Dermatology and Venereology, HIV Outpatient Clinic, University Hospital Essen, Essen, Germany

^3^ Interdisciplinary Immunological Outpatient Clinic, Center for Sexual Health and Medicine, Department of Dermatology, Venereology and Allergology, Ruhr University Bochum, Bochum, Germany

^4^ Department of Pneumology, Infectious Diseases and Internal Medicine, Klinikum Dortmund, Hospital University Witten/ Herdecke, Dortmund, Germany

^5^ Department of Infectious Diseases, University Hospital Essen, Essen, Germany

^6^ Institute for translational HIV research, University Hospital Essen, Essen, Germany

**Corresponding author:**

Stefan Esser, MD, Clinic for Dermatology and Venerology, University Hospital Essen, University of Duisburg-Essen, Hufelandstr. 55, Essen 45122, Germany

Telephone: +49 201 723 3878, Fax.: +49 201 723 3845, e-mail: stefan.esser@uk-essen.de


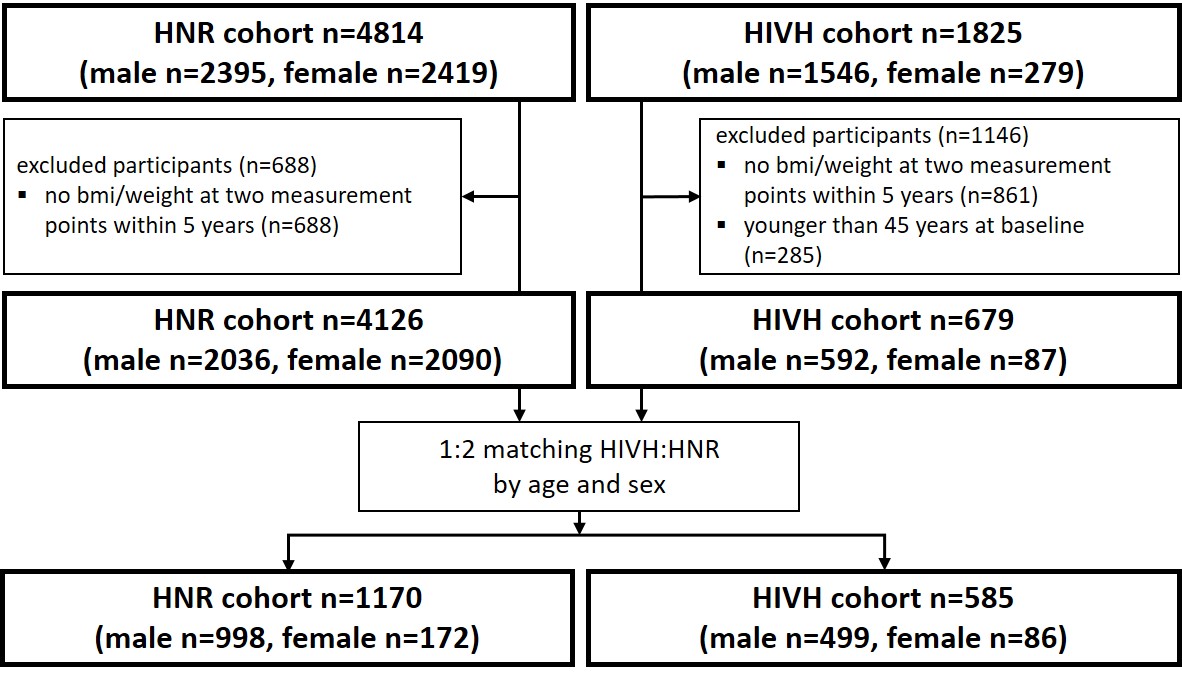


Online Resource 1: flowchart patients of HIVH & HNR included in analysis matched 1:2 by age and sex (state HIVH June 2021)


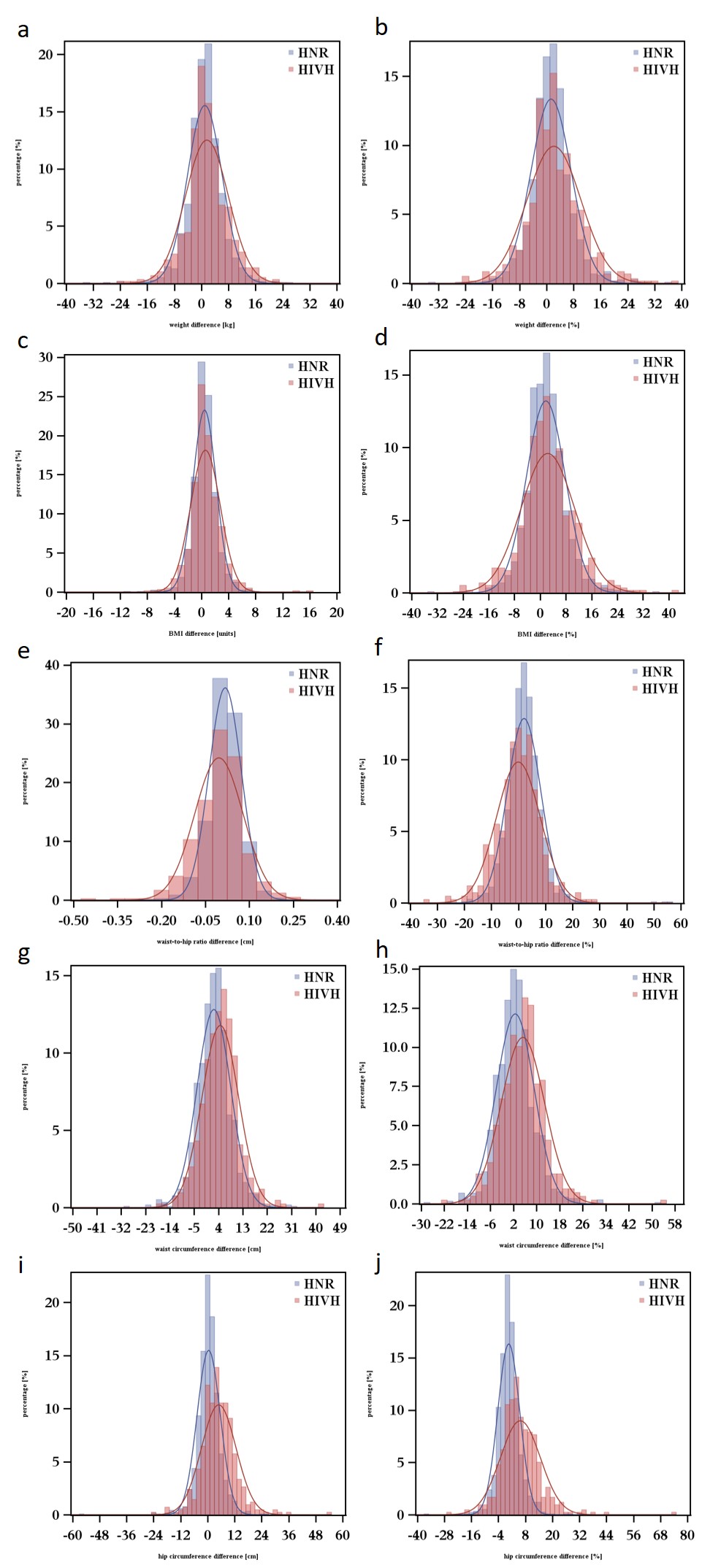


Online Resource 2: Histograms for the absolute and relative weight (a & b), BMI (c & d), WHR (e & f), waist circumference (g & h), and hip circumference (i & j) difference within 5 years in HIVH and HNR

Online Resource 3: absolute and relative weight gain in HIVH and HNR

| **study** | **weight gain (relative)** | | | |
| --- | --- | --- | --- | --- |
|  | **≤-10%** | **>-10% to <10%** | **≥10%** | **total** |
| **HIVH** | 34 (5.8 %) | 472 (80.7 %) | 79 (13.5 %) | 585 |
| **HNR** | 33 (2.8 %) | 1064 (90.9 %) | 73 (6.2 %) | 1170 |
|  | **weight gain (absolute)** | | | |
|  | **≤-10kg** | **>-10% to <10%** | **≥10kg** | **total** |
| **HIVH** | 20 (3.4 %) | 519 (88.7 %) | 46 (7.9 %) | 585 |
| **HNR** | 23 (2 %) | 1104 (94.4 %) | 43 (3.7 %) | 1170 |

Online Resource 4: HIVH & HNR characteristics stratified by relative weight gain

|  | | **HIVH** | | **HNR** | | **HIVH** | **HNR** |
| --- | --- | --- | --- | --- | --- | --- | --- |
|  |  | **weight change within 5 years ≥10%** | | | | | |
|  |  | **Baseline** | | | | **absolute change after 5 years** | |
|  |  | **N** | **n (%) / MEAN ± SD** | **N** | **n (%) / MEAN ± SD** | **n %) / MEAN ± SD** | **n (%) / MEAN ± SD** |
| **sex** | **female** | 79 | 17 (21.52 %) | 73 | 26 (35.6 %) |  | |
| **age** | **[years]** | 79 | 51.94 ± 4.8 | 73 | 52.68 ± 5.45 |  |  |
| **blood pressure** | **systolic [mmHg]** | 69 | 139.77 ± 17.73 | 73 | 130.23 ± 20.53 | -2.74 ± 23.05 | +5.96 ± 19.58 |
|  | **diastolic [mmHg]** | 69 | 85.56 ± 12.19 | 73 | 81.09 ± 10.79 | -1.78 ± 11.67 | +1.09 ± 11.75 |
| **diabetes mellitus** | **yes** | 77 | 2 (2.6 %) | 73 | 10 (13.7 %) | +3 (4 %) | +8 (12.7 %) |
| **total cholesterol** | **[mg/dl]** | 76 | 204.29 ± 43.9 | 73 | 217.82 ± 35.68 | +8.96 ± 45.27 | +9.77 ± 39.97 |
| **LDL** | **[mg/dl]** | 71 | 125.33 ± 37.4 | 73 | 132.9 ± 31.67 | +14.82 ± 39.74 | +3.31 ± 30.69 |
| **HDL** | **[mg/dl]** | 73 | 50.31 ± 18.72 | 73 | 55.11 ± 16.9 | -0.77 ± 12.49 | +1.13 ± 10.2 |
| **weight** | **[kg]** | 79 | 77.11 ± 14.18 | 73 | 79.02 ± 14.05 | +11.99 ± 4.88 | +11.51 ± 3.97 |
| **BMI** | **[kg/m^2^]** | 79 | 25.38 ± 3.94 | 73 | 27.13 ± 4.27 | +4.05 ± 2.24 | +4.04 ± 1.28 |
| **waist-to-hip ratio** |  | 67 | 0.98 ± 0.07 | 73 | 0.93 ± 0.09 | +0.01 ± 0.11 | +0.05 ± 0.05 |
| **smoking** | **yes** | 67 | 38 (56.72 %) | 73 | 37 (50.68 %) | +1 (9.09 %) | 0 |
|  |  | **weight change within 5 years <10% to >-10%** | | | | | |
|  |  | **Baseline** | | | | **absolute change after 5 years** | |
| **sex** | **female** | 509 | 71 (13.95 %) | 1117 | 164 (14.68 %) |  | |
| **age** | **[years]** | 509 | 54.64 ± 6.82 | 1117 | 54.8 ± 6.76 |  |  |
| **blood pressure** | **systolic [mmHg]** | 459 | 139.03 ± 19.28 | 1116 | 133.51 ± 19.13 | -1.2 ± 20.08 | +1.2 ± 18.2 |
|  | **diastolic [mmHg]** | 459 | 84.22 ± 11.79 | 1116 | 83.53 ± 10.85 | -2.18 ± 11.84 | -2.22 ± 10.84 |
| **diabetes mellitus** | **yes** | 509 | 44 (9.05 %) | 1117 | 146 (13.07 %) | +21 (4.75 %) | +87 (8.96 %) |
| **total cholesterol** | **[mg/dl]** | 502 | 212.67 ± 42.72 | 1113 | 225.09 ± 37.6 | +4.9 ± 44.97 | -3.72 ± 36.34 |
| **LDL** | **[mg/dl]** | 455 | 130.14 ± 38.66 | 1110 | 143.61 ± 35.17 | +10.3 ± 39.7 | -11.9 ± 31.2 |
| **HDL** | **[mg/dl]** | 473 | 49.78 ± 15.79 | 1112 | 52.96 ± 15.12 | +1.75 ± 11.27 | +1.77 ± 9.88 |
| **weight** | **[kg]** | 509 | 77.76 ± 14.57 | 1117 | 83.8 ± 14.15 | +2.64 ± 5.67 | +1.45 ± 4.47 |
| **BMI** | **[kg/m^2^]** | 509 | 25.1 ± 4.21 | 1117 | 27.58 ± 3.93 | +0.92 ± 2 | +0.61 ± 1.51 |
| **waist-to-hip ratio** |  | 420 | 0.98 ± 0.07 | 1117 | 0.95 ± 0.08 | +0 ± 0.08 | +0.02 ± 0.05 |
| **smoking** | **yes** | 435 | 191 (43.91 %) | 1117 | 320 (28.65 %) | +12 (29.27 %) | +30 (27.03 %) |
|  |  | **weight change within 5 years ≤-10%** | | | | | |
|  |  | **Baseline** | | | | **absolute change after 5 years** | |
| **sex** | **female** | 34 | 7 (20.59 %) | 33 | 5 (15.15 %) |  | |
| **age** | **[years]** | 34 | 56.91 ± 6.35 | 33 | 55.91 ± 7.98 |  |  |
| **blood pressure** | **systolic [mmHg]** | 28 | 137.89 ± 16.72 | 32 | 137.78 ± 18.68 | -6.69 ± 21.58 | -4.82 ± 18.13 |
|  | **diastolic [mmHg]** | 28 | 80.89 ± 10.2 | 32 | 83.89 ± 9.64 | -2.47 ± 15.47 | -6.13 ± 9.19 |
| **diabetes mellitus** | **yes** | 32 | 4 (12.5 %) | 33 | 7 (21.21 %) | +4 (14.29 %) | +4 (15.38 %) |
| **total cholesterol** | **[mg/dl]** | 34 | 211.18 ± 47.1 | 33 | 222.88 ± 42.25 | -14.97 ± 73.69 | -20.5 ± 35.31 |
| **LDL** | **[mg/dl]** | 29 | 128.59 ± 42.63 | 32 | 152.69 ± 41.05 | -3.16 ± 60.22 | -27.79 ± 34.6 |
| **HDL** | **[mg/dl]** | 31 | 50.31 ± 15.96 | 33 | 46.7 ± 16.47 | +0.47 ± 12.66 | +3.37 ± 8.66 |
| **weight** | **[kg]** | 34 | 81.09 ± 17.1 | 33 | 94.92 ± 20.15 | -11.85 ± 4.27 | -13.52 ± 6.2 |
| **BMI** | **[kg/m^2^]** | 34 | 26.59 ± 4.65 | 33 | 31.37 ± 5.96 | -3.8 ± 1.32 | -4.24 ± 2.01 |
| **waist-to-hip ratio** |  | 23 | 0.99 ± 0.07 | 32 | 0.98 ± 0.07 | +0 ± 0.09 | -0.02 ± 0.07 |
| **smoking** | **yes** | 25 | 12 (48 %) | 33 | 11 (33.33 %) | 0 | +2 (66.67 %) |
