# Supplementary material for: Changes in body mass index, weight, and waist-to-hip ratio over five years in HIV-positive individuals in the HIV Heart Aging Study compared to the general population
Source: Infection. 2023 Mar 17;51(4):1081–91. doi: 10.1007/s15010-023-02009-8 (PMC10352154; doi:10.1007/s15010-023-02009-8)
Supplement: Supplementary file 2 — Supplementary file2 (PDF 233 KB) [file 15010_2023_2009_MOESM2_ESM.pdf]

Changes in body mass index, weight, and waist-to-hip ratio over five years in HIV-positive individuals in the HIV Heart Aging Study compared to the general population

Infection

Authors:

Laven Mavarani[1], Sarah Albayrak-Rena[2], Anja Potthoff[3], Martin Hower[4], Sebastian Dolff[5], Stefanie Sammet[2], Felix Maischack[2], Dirk Schadendorf[2], Börge Schmidt[1], Stefan Esser[2,6] on behalf of HIV HEART AGING Study

Affiliations:

[1] Institute for Medical Informatics, Biometry and Epidemiology (IMIBE), University Hospital Essen, Essen, Germany

[2] Department of Dermatology and Venereology, HIV Outpatient Clinic, University Hospital Essen, Essen, Germany

[3] Interdisciplinary Immunological Outpatient Clinic, Center for Sexual Health and Medicine, Department of Dermatology, Venereology and Allergology, Ruhr University Bochum, Bochum, Germany

[4] Department of Pneumology, Infectious Diseases and Internal Medicine, Klinikum Dortmund, Hospital University Witten/ Herdecke, Dortmund, Germany

[5] Department of Infectious Diseases, University Hospital Essen, Essen, Germany

[6] Institute for translational HIV research, University Hospital Essen, Essen, Germany

Corresponding author:

Stefan Esser, MD, Clinic for Dermatology and Venerology, University Hospital Essen, University of Duisburg-Essen, Hufelandstr. 55, Essen 45122, Germany

Telephone: +49 201 723 3878, Fax.: +49 201 723 3845, e-mail: stefan.esser@uk-essen.de

Online Resource 5: HIVH HIV-specific characteristics stratified by relative weight change within 5 years of ≥10%, <10% to >-10%, and ≤-10%

		HIVH weight change within 5 years ≥10%				HIVH weight change within 5 years <10% to >-10%				HIVH weight change within 5 years ≤-10%			
		Baseline		after 5 years		Baseline		after 5 years		Baseline		after 5 years	
		N	n (%) / MEAN ± SD	N	n (%) / MEAN ± SD	N	n (%) / MEAN ± SD	N	n (%) / MEAN ± SD	N	n (%) / MEAN ± SD	N	n (%) / MEAN ± SD
**age at HIV-infection**	**[years]**	78	38.7 ± 7.2	78	38.7 ± 7.2	499	40.6 ± 9.3	499	40.6 ± 9.3	33	41.8 ± 9.9	33	41.8 ± 9.9
**ethnicity**	**caucasian**	79	69 (87.3 %)	79	69 (87.3 %)	509	469 (92.1 %)	509	469 (92.1 %)	34	33 (97.1 %)	34	33 (97.1 %)
	**african**	79	2 (2.5 %)	79	2 (2.5 %)	509	8 (1.6 %)	509	8 (1.6 %)	34	0	34	0
	**asian**	79	2 (2.5 %)	79	2 (2.5 %)	509	4 (0.8 %)	509	4 (0.8 %)	34	1 (2.9 %)	34	1 (2.9 %)
	**other**	79	6 (7.6 %)	79	6 (7.6 %)	509	28 (5.5 %)	509	28 (5.5 %)	34	0	34	0
**way of HIV transmission**	**MSM**	75	39 (52.0 %)	75	39 (52.0 %)	483	309 (64.0 %)	483	309 (64.0 %)	34	23 (67.7 %)	34	23 (67.7 %)
	**hetero**	75	24 (32.0 %)	75	24 (32.0 %)	483	104 (21.5 %)	483	104 (21.5 %)	34	9 (26.5 %)	34	9 (26.5 %)
	**intravenous drug use**	75	5 (6.7 %)	75	5 (6.7 %)	483	33 (6.8 %)	483	33 (6.8 %)	34	2 (5.9 %)	34	2 (5.9 %)
	**transfusion**	75	2 (2.7 %)	75	2 (2.7 %)	483	10 (2.1 %)	483	10 (2.1 %)	34	0	34	0
	**epidemic area**	75	5 (6.7 %)	75	5 (6.7 %)	483	27 (5.6 %)	483	27 (5.6 %)	34	0	34	0
	**other**	75	0	75	0	483	0	483	0	34	0	34	0
**HIV categorie (immonological)**	**HIV I**	77	3 (3.9 %)	78	2 (2.6 %)	484	21 (4.3 %)	508	14 (2.8 %)	32	1 (3.1 %)	34	1 (2.9 %)
	**HIV II**	77	39 (50.7 %)	78	39 (50.0 %)	484	212 (43.8 %)	508	216 (42.5 %)	32	10 (31.3 %)	34	11 (32.4 %)
	**HIV III**	77	35 (45.5 %)	78	37 (47.4 %)	484	251 (51.9 %)	508	278 (54.7 %)	32	21 (65.6 %)	34	22 (64.7 %)

**HIV categorie (clinical)**	**HIV A**	77	32 (41.6 %)	78	31 (39.7 %)	484	159 (32.9 %)	508	147 (28.9 %)	32	9 (28.1 %)	34	9 (26.5 %)
	**HIV B**	77	19 (24.7 %)	78	20 (25.6 %)	484	160 (33.1 %)	508	174 (34.3 %)	32	9 (28.1 %)	34	9 (26.5 %)
	**HIV C / AIDS**	77	26 (33.8 %)	78	27 (34.6 %)	484	165 (34.1 %)	508	187 (36.8 %)	32	14 (43.8 %)	34	16 (47.1 %)
**CD4/CD8 ratio**		79	0.82 ± 0.42	79	0.88 ± 0.41	504	0.8 ± 0.44	505	1.02 ± 2.11	33	0.83 ± 0.41	33	0.92 ± 0.6
**viral load**	**[copies/ml]**	79	46.9 ± 107.5	79	36.5 ± 65.9	509	707.3 ± 10365.8	508	213.7 ± 3945.8	34	4382.5 ± 25372.9	34	44.3 ± 104.8
**viral load < 50 copies/ml**	**yes**	79	73 (92.4 %)	79	72 (91.1 %)	509	474 (93.1 %)	509	479 (94.1 %)	34	31 (91.2 %)	34	32 (94.12%)
**duration of HIV infection**	**[years]**	78	13.2 ± 6.2	78	18.5 ± 6.2	499	14.1 ± 6.7	499	19.3 ± 6.7	33	14.9 ± 6.9	33	19.8 ±7.0
**ART medication categories**	**NRTIs**	78	74 (94.9 %)	79	72 (91.1 %)	498	468 (94.0 %)	501	452 (90.2 %)	33	31 (93.9 %)	34	33 (97.1 %)
	**NRTIs:TAF**	78	0	79	34 (43.0 %)	498	5 (1 %)	501	210 (41.9 %)	33	0	34	9 (26.47 %)
	**NNRTIs**	78	33 (42.3 %)	79	21 (26.6 %)	498	235 (47.2 %)	501	192 (38.3 %)	33	12 (36.4 %)	34	10 (29.4 %)
	**INSTIs**	78	12 (15.4 %)	79	51 (64.6 %)	498	73 (14.7 %)	501	234 (46.7 %)	33	3 (9.1 %)	34	11 (32.4 %)
	**PIs**	78	39 (50.0 %)	79	15 (19.0 %)	498	238 (47.8 %)	501	141 (28.1 %)	33	18 (54.5 %)	34	17 (50.0 %)
	**boost**	78	28 (35.9 %)	79	18 (22.8 %)	498	174 (34.9 %)	501	155 (30.9 %)	33	10 (30.3 %)	34	12 (35.3 %)
	**entry inhibitor**	78	3 (3.8 %)	79	3 (3.8 %)	498	13 (2.6 %)	501	15 (3.0 %)	33	1 (3.0 %)	34	2 (5.9 %)
	**combination medication**	78	55 (70.5 %)	79	29 (36.7 %)	498	355 (71.3 %)	501	231 (46.1 %)	33	24 (72.7 %)	34	20 (58.8 %)
	**single tablet regime**	78	15 (19.2%)	79	41 (51.7 %)	498	82 (16.5 %)	501	208 (41.5 %)	33	3 (9.1 %)	34	8 (23.5 %)
**Lipodystrophy**	**yes**	75	13 (17.3 %)	79	17 (21.5 %)	478	101 (21.1 %)	508	115 (22.6 %)	31	10 (32.3 %)	34	9 (26.5 %)
**Lipoatrophy**	**yes**	76	9 (11.8 %)	79	10 (12.7 %)	480	109 (22.7 %)	509	108 (21.2 %)	31	5 (16.1 %)	34	7 (20.6 %)
**Lipohypertrophy**	**yes**	76	7 (9.2 %)	78	5 (6.4 %)	415	50 (10.4 %)	508	43 (8.5 %)	31	7 (22.6 %)	34	6 (17.7 %)
